# Antimicrobial Resistance Profile of *Acinetobacter* spp. Isolates from Retail Meat Samples under *Campylobacter*-Selective Conditions

**DOI:** 10.4014/jmb.2102.02027

**Published:** 2021-04-05

**Authors:** Min-Hyeok Cha, Sun Hee Kim, Seokhwan Kim, Woojung Lee, Hyo-Sun Kwak, Young-Min Chi, Gun-Jo Woo

**Affiliations:** 1 Laboratory of Food Safety and Evaluation, Department of Biotechnology, Korea University Graduate School, Seoul 02841, Republic of Korea; 2 Division of Biotechnology, College of Life Sciences and Biotechnology, Korea University, Seoul 02841, Republic of Korea; 3 Division of Food Microbiology, National Institute of Food and Drug Safety Evaluation, Ministry of Food and Drug Safety, Cheongju 28159, Republic of Korea; 4 Department of Food Science and Biotechnology, Kyung Hee University, Gyeonggi-do 17104, Republic of Korea

**Keywords:** *Acinetobacter*, meat, antimicrobial resistance, food surveillance, colistin resistance

## Abstract

*Acinetobacter* strains are widely present in the environment. Some antimicrobial-resistant strains of this genus have been implicated in infections acquired in hospitals. Genetic similarities have been reported between *Acinetobacter* strains in nosocomial infections and those isolated from foods. However, the antimicrobial resistance of *Acinetobacter* strains in foods, such as meat, remains unclear. This study initially aimed to isolate *Campylobacter* strains; instead, strains of the genus *Acinetobacter* were isolated from meat products, and their antimicrobial resistance was investigated. In total, 58 *Acinetobacter* strains were isolated from 381 meat samples. Of these, 32 strains (38.6%) were from beef, 22 (26.5%) from pork, and 4 (4.8%) from duck meat. Antimicrobial susceptibility tests revealed that 12 strains were resistant to more than one antimicrobial agent, whereas two strains were multidrug-resistant; both strains were resistant to colistin. Cephalosporin antimicrobials showed high minimal inhibitory concentration against *Acinetobacter* strains. Resfinder analysis showed that one colistin-resistant strain carried *mcr-4.3*; this plasmid type was not confirmed, even when analyzed with PlasmidFinder. Analysis of the contig harboring *mcr-4.3* using BLAST confirmed that this contig was related to *mcr-4.3* of *Acinetobacter baumannii*. The increase in antimicrobial resistance in food production environments increases the resistance rate of *Acinetobacter* strains present in meat, inhibits the isolation of *Campylobacter* strains, and acts as a medium for the transmission of antimicrobial resistance in the environment. Therefore, further investigations are warranted to prevent the spread of antimicrobial resistance in food products.

## Introduction

*Acinetobacter baumannii*, a gram-negative opportunistic pathogen that causes multidrug-resistant infections, can grow under conditions selective for *Campylobacter* spp. [1]. Members of the genus *Acinetobacter* are typically found in the soil, where they play an important role in mineralization and organic compound decomposition for plant use [2]. However, in recent years, the genus *Acinetobacter*, particularly multidrug-resistant *A. baumannii*, has become a major causative agent of opportunistic infections. These bacteria, found in emergency rooms and intensive care units, are responsible for various infectious diseases such as pneumonia, wound tissue infection, sepsis, and urinary tract infection [2].

Recently, genetic similarities have been reported between the strains responsible for human infection in hospitals and *A. baumannii* isolated from food [3]. In addition, infections caused by colistin-resistant *Acinetobacter* have been reported [4]. In gram-negative pathogenic bacteria, such as *Acinetobacter*, the emergence of a strain carrying mobile colistin resistance (*mcr*) gene, which induces colistin resistance, is a major global health challenge [5]. Therefore, the spread of the colistin-resistant gene in various environments must be monitored and restricted [6]. Specifically, understanding the overall status of antimicrobial resistance in the food chain is important for identifying transmission pathways for resistant bacteria and blocking their transmission [7].

In this regard, the antimicrobial resistance of food-derived *A. baumannii* and its molecular dynamics requires special attention. Several studies performed in Korea have shown that *A. baumannii* causes infections in hospitals; however, data regarding the presence and antimicrobial resistance of *A. baumannii* on foods, such as meat distributed in the market, remain limited. This study initially aimed to isolate and characterize *Campylobacter*, including antimicrobial resistance; however, we instead isolated *Acinetobacter* strains since they were more abundant. Despite this change in study aim, our results addressed several insufficiencies reported by previous studies.

In the present study, we isolated the strains of the genus *Acinetobacter* grown in a *Campylobacter*-specific environment and identified them using molecular methods to confirm their distribution rate and antimicrobial resistance status. We also selected a colistin-resistant strain and performed next-generation sequencing (NGS) analysis to compare the sequence similarities with those of strains isolated from the clinical environment.

## Materials and Methods

### Sample Collection

In the present study, we collected five types of meat and meat products. The sample types were selected based on their consumption rates. The samples comprised domestic and imported meat products, including beef, pork, chicken, duck, and aquatic products. These samples (number of samples indicated in square brackets, number of domestic and imported products are separated with a comma, respectively) included beef [*n* = 59, 60], pork [*n* = 70, 37], chicken meat [*n* = 83 (only domestic)], duck meat [*n* = 42 (only domestic), and aquatic products [*n* = 10, 20]. In total, 381 samples (domestic meat [*n* = 264] and imported meat [*n* = 117]) were collected from 13 hypermarkets in Korea between February and October 2019. Samples were transported under refrigerated conditions within 24 h of collection to the laboratory for analysis. The sample distribution is shown in Table 1.

### Isolation of *Acinetobacter* spp.

Acinetobacter spp. were isolated as previously described in the Korean Food Code 2020 for *Campylobacter* spp. isolation [8]. Twenty-five grams of each meat sample was inoculated into 225ml of Bolton medium (Oxoid, UK) and cultured at 42°C under microaerophilic conditions (5% O_2_, 85% N_2_, and 10% CO_2_) for 48h. Next, the cultures were spread on modified Charcoal-Cefoperazone-Deoxycholate Agar (Oxoid) and incubated at 42°C under microaerophilic conditions for 24h. Subsequently, three or more colonies with a circular or irregular shape and translucent or transparent in appearance in grayish-white tones were selected and identified using the VITEK-MS system (BioMérieux Vitek, France). The isolates, identified as belonging to “A. baumannii complex,” were selected for further molecular characterization.

### Identification of *A. baumannii*

The collected strains were identified using molecular and microbiological methods. First, collected strains were cultured in trypticase soy agar (Oxoid) for 24 h. The cultured colonies were suspended in 200 μl of sterile distilled water to obtain genomic DNA, followed by amplification and sequencing of two segments of the 16S rRNA (27F: 5'-AGA GTT TGA TCC TGG CTC AG-3', 1492R: 5'-TAC GGY TAC CTT GTT ACG ACT T-3'), rpoB zone 1 (Ac696F: 5'-TAY CGY AAA GAY TTG AAA GAA G-3', Ac1093R: 5'-MAC ACC YTT GTT MCC RTG A-3') and rpoB zone 2 (Ac1055F: 5'-GTG ATA ARA TGG CBG GTC GT-3', Ac1598R: 5'-CGB GCR TGC ATY TTG TCR T-3'). The 16S rRNA gene was compared to the Eztaxon database of ChunLab (https://www.ezbiocloud.net/) to confirm the high agreement (sequence identity of > 98.7%), whereas, for *rpoB*, zones 1 and 2 (two variable areas bordered by highly conserved regions in *rpoB*. For zone 1, between positions 2,900 and 3,250, while for zone 2, between positions 3,250 and 3,700) were sequenced and compared via BLAST analysis [9].

### Antimicrobial Susceptibility Tests

Antimicrobial susceptibility tests were performed using Sensititre KRNVF5F panels (Trek Diagnostic Systems, USA) to measure the MIC value by the broth microdilution method. The following 16 antimicrobials were used to evaluate the antimicrobial susceptibility of the isolated strains: amoxicillin/clavulanic acid (2:1), cefoxitin, chloramphenicol, ciprofloxacin, gentamicin, tetracycline, nalidixic acid, meropenem, cefepime, colistin, trimethoprim/sulfamethoxazole, ampicillin, ceftazidime, sulfisoxazole, ceftiofur, and streptomycin. The breakpoint of the antimicrobials was decided based on the CLSI guidelines [10]. However, no resistance criteria have been provided in the CLSI guidelines for the following antimicrobials: amoxicillin/clavulanic acid (2:1), cefoxitin, chloramphenicol, nalidixic acid, ampicillin, sulfisoxazole, ceftiofur, and streptomycin. For these agents, the degree of increase in resistance was determined by referring to the resistance level of the standard strain, ATCC 17978 [6].

### NGS and Bioinformatics Analyses

Colistin-resistant *Acinetobacter* spp. isolates were subjected to NGS (Macrogen, Korea), and the genome sequences of these strains were obtained using the Illumina HiSeqXten platform (USA). The acquired antimicrobial resistant gene and plasmid replicon typing were identified *in silico* using ResFinder 3.2 and the PlasmidFinder 2.1 webserver (https://cge.cbs.dtu.dk, accessed June 24, 2020), respectively [11, 12]. The genome was annotated using RAST (http://rast.theseed.org/) to analyze the genetic environment of *mcr* [13]. BLAST was used to align the genetic sequences flanking *mcr* (www.ncbi.nlm.nih.gov/BLASTih.gov/BL). The results were visualized using SnapGene (GSL Biotech; available at snapgene.com). ISfinder was used to check the presence and type of the insertion sequence in a contig (database URL, http://www-is.biotoul.fr) [14].

## Results

### Isolation and Identification of *Acinetobacter* spp.

Overall, 58 strains (15.2%) of *Acinetobacter* spp. were isolated from 381 samples using the *Campylobacter* growth environment, identified with Vitek-MS IVD mode. A total of 32 strains (55.2%) were isolated from beef, 22 (37.9%) from pork, and 4 (6.9%) from duck meat; no strains were isolated from chicken meat and aquatic products. Sequence analysis of 16S rRNA and *rpoB* from the isolated strains showed that 33 of the isolated strains exhibited sequence homology with *A. baumannii*. In contrast, 17 strains showed sequence homology to *Acinetobacter nosocomialis*, 7 strains to *Acinetobacter seifertii*, and one strain to *Acinetobacter pittii* (Table 1). This result was used as a reference for species identification.

### Antimicrobial Resistance Profile of *Acinetobacter* Isolates

The antimicrobial susceptibility test results of the 58 isolates are summarized in Tables 2 and 3. Of the 16 antimicrobials tested, the isolates were resistant to only five types of antimicrobials. The isolates were the most resistant to trimethoprim/sulfamethoxazole, with 10 isolates being resistant to this drug combination. Three isolates were resistant to gentamicin and tetracycline; only two isolates were resistant to colistin, and one isolate was resistant to cefepime. The MICs of the two isolates resistant to colistin were 16 μg/ml (*A. nosocomialis*, KUFSE-ACN036) and 4 μg/ml (*A. seifertii* KUFSE-ACS055), respectively.

Two isolates were resistant to three antimicrobials, one isolate was resistant to two antimicrobials, while the remaining nine isolates were resistant to only one antimicrobial, as shown in Table 3. The resistant isolates included *A. nosocomialis* (*n* = 6), *A. baumannii* (*n* = 3), and *A. seifertii* (*n* = 3). The results of antimicrobial susceptibility analysis are summarized in Table 3.

### Detection of *mcr-4.3* and *in silico* Molecular Characteristic Analysis

Table 4 shows the ResFinder and PlasmidFinder analysis results for the colistin-resistant strain (KUFSE-ACN036, MIC: 16 μg/ml). This colistin-resistant strain (*A. nosocomialis* KUFSE-ACN036) harbored *mcr-4.3*, while the other colistin-resistant strain (KUFSE-ACS055) did not harbor any *mcr* genes. However, these strains did not carry any other antimicrobial resistance genes.

### Genetic Characterization of Colistin Resistance Strains

**Fig. 1:**
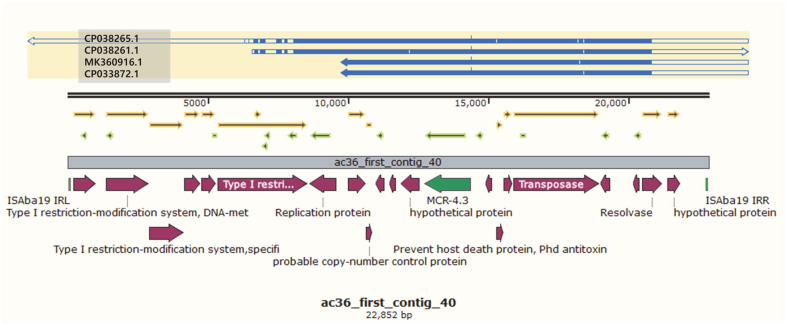
Sequence alignment of the top four BLAST search results in the full part of contig 40 of KUFSEACN036. Blue arrows indicate the degree and orientation of the reference genes (CP038265.1, CP038261.1, MK360916.1, and CP033872.1). The light green square indicates the insertion sequence IS*Aba19*. The green arrow indicates *mcr-4.3*. Red arrows indicate other genes. Arrows highlighted in yellow and light green indicate open reading frames. Sequence comparison, alignment, and drawing were performed using SnapGene 5.0.8.

The results of contig 40 analysis of KUFSE-ACN036 harboring *mcr-4.3* using BLAST showed that the top 4 matches based on the maximum score carried plasmids derived from *A. baumannii* (Table 5). Among them, the strain with accession number CP038265.1 showed the highest maximum score of 26,502. This strain carried the *A. baumannii* plasmid, which was previously reported to possess *mcr-4.3*. The remaining top three strains carried plasmids with *mcr-4.3* (CP038261.1, MK360916.1, and CP033872.1); however, their query covers were only 63–80%; therefore, no plasmids in GenBank matched all contig 40. Analysis using ISfinder showed that contig 40 was related to IS*Aba19*. Although not all these sequences were available, the sequences of IS*Aba19* IRL (5′-TGA ACC GTA CCG GGT TTG TCG GAG AGT CAA TAT TCT GAG AGA CTA TCC CG-3′) and IRR (5′-TGA ACC GTA CCG GGT TTG TCG GAG ACT TTT TTA TTT AAG TTA AGC CAC CT-3′) were included in contig 40. Contig 40 has a total length of 21,130 base pairs, in which CP03261.1 matched over a specific part (6702..21,130) in the middle (Fig. 1). As BLAST analysis was performed on both ends (1..6701 and 21,131..22,852) of contig 40 (except for the middle portion), the match with other bacteria included in the *Acinetobacter* spp. was confirmed. Interestingly, the top matched gene sequences of each two-part BLAST results were the same (CP028138.1, CP044018.1, CP046595.1, and CP044445.1) (Table 6).

## Discussion

We successfully isolated *Acinetobacter* genus strains from commercial meat products using conditions selective for *Campylobacter* isolation. Microbiological and molecular methods were used to characterize the isolates to the species level; the characterized strains included *A. nosocomialis*, *A. pittii*, and *A. seifertii*, in addition to *A. baumannii*. To the best of our knowledge, no previous study has reported the isolation of strains belonging to the genus *Acinetobacter* from food using the microbiological and molecular methods discussed in the present study. Although a previous study by Fernando *et al*. [1] reported the isolation of *A. baumannii* from environmental reservoirs, its presence in meat products openly sold in retail markets has not been studied. Due to the availability and large-scale consumption of meat products, combined with the environmental transmission of *mcr*, it is important to identify bacterial isolates present in meat products.

This study used mCCDA as a selection medium, while the modified Karmali agar was used by Fernando *et al*.[1]. However, both media contained antimicrobials that increased the specificity of isolating *Campylobacter* (modified Karmali agar: cefoperazone-32 mg/l, vancomycin-20 mg/l, and amphotericin B-10 mg/l; mCCDA: cefoperazone-32 mg/l, and amphotericin B-10 mg/l) [1]. Since *Campylobacter* is naturally resistant to these antimicrobials, they were added to inhibit the growth of other strains. However, in this study, *Acinetobacter* spp. could grow in media containing these antimicrobials, indicating that the resistance of *Acinetobacter* spp., which can be isolated from the environment, has increased [15, 16]. Thus, it is presumed that these resistance factors were disseminated to *Acinetobacter* strains in the environment. In this study, no resistant strains were found in poultry meat, while 6 resistant strains were isolated from pork and beef. There is a difference in antimicrobials used depending on the type of livestock. Further studies on *Acinetobacter* strains in livestock are warranted to better understand the migration pattern of this resistance factor.

Antimicrobial resistance genes spread in livestock environment can be continuously transmitted between other bacteria [17]. Originally, this study aimed to isolate only *Campylobacter* bacteria under selective conditions; however, only two *C. jejuni* and three *C. coli* strains were isolated from chicken and duck meat (data not shown here). This result suggests that using a selective media that can inhibit the growth of certain bacteria while allowing the growth of others is no longer sufficient due to the increasing number of resistant strains. For instance, the growth of *Acinetobacter* bacteria, resistant to these antimicrobials, can suppress the growth or selection of *Campylobacter* under *Campylobacter*-specific culture conditions.

A high rate of *Acinetobacter* isolation can be achieved from various raw meat and agricultural products using CHROMagar and Dijkshoorn's media [18, 19], which can be used to isolate *Acinetobacter*. Additionally, *Acinetobacter* is intrinsically resistant to cephalosporin-based antimicrobials. Therefore, to effectively isolate *Campylobacter*, it is necessary to consider the differential biochemical properties of target strains apart from antimicrobial resistance profile.

*Acinetobacter* is commonly present in the environment, and the transmission of antimicrobial resistance can increase the number of antimicrobial-resistant strains in our environment and the food we consume. Particularly, an increase in antimicrobial resistance hampers clinical treatment, and consumption of food items infested by these resistant strains poses serious risks to immunocompromised individuals [20]. Therefore, studies on the presence and prevalence of *Acinetobacter* strains in food products are urgently needed.

The colistin-resistant gene can spread horizontally, and the genus *Acinetobacter* plays a key role in carrying and disseminating mobile colistin resistance genes in the environment. These are known as *mcr*-gene-containing bacteria, which have been propagated by horizontal/lateral transfer to various ecosystems, including wild animals or livestock; environments such as soil, stream water, plant or agricultural systems; and human communities [21]. In the present study, *mcr-4.3* was identified in only one strain. To the best of our knowledge, this is the first study to report the isolation of a strain belonging to the genus *Acinetobacter* from meats in Korean food hypermarkets. Moreover, the strain (KUFSE-ACN036) was isolated from pork imported from the Netherlands. Although no *Acinetobacter* strain isolated from domestic meat showed resistance to colistin, further research is needed to investigate the distribution of *mcr* genes in *Acinetobacter* strains from domestic livestock farms. *mcr-4* was first reportedly isolated in Italy, while the *mcr-4.3* genotype has been widely reported in Europe, which is consistent with our study results [22-24]. However, recent studies have reported this gene in countries outside Europe, including China and Brazil [5, 25]. The path of dissemination of antimicrobial resistance warrants further investigation.

BLAST analysis results showed that *mcr-4.3* identified in this study is related to *A. baumannii*. However, there is no complete alignment in BLAST results with contig 40. The aligned part containing *mcr-4.3* showed results consistent with the plasmids, while the other parts (both ends) showed results consistent with chromosomes. No plasmid type was identified through PlasmidFinder, suggesting that the plasmid containing *mcr-4.3* identified in the present study is novel. The entire plasmid remains to be confirmed because NGS analysis was based on the short-read method, which is a limitation of the present study. Therefore, further studies are warranted to identify the complete sequence of the plasmid using long-read sequencing; epidemiological studies based on molecular biological methods can provide deeper insights into antimicrobial resistance transmission.

In conclusion, we isolated *Acinetobacter* bacteria from culture conditions specific for *Campylobacter* isolation. Further, we confirmed the antimicrobial resistance properties and molecular epidemiological characteristics of the isolated strains, in addition to the presence of the colistin resistance gene. This study suggests the transmission of antimicrobial resistance to microbes commonly present in our environments and food products and highlights the imminent threat posed by the increased number of infections caused by these microbes. Moreover, their multidrug resistance profile may limit therapeutic options. Therefore, continuous efforts are needed to prevent the spread of antimicrobial resistance by judicious use of antimicrobials and identifying novel microbial agents to treat multidrug-resistant strains.

## Figures and Tables

**Table 1:** Distribution and identification of *Acinetobacter* spp. isolated from meat samples using *Campylobacter*selective conditions.

Species	Beef		Pork		Chicken meat	Duck meat	Aquatic product

Number of samples	119		107		83	42	30

Sample origin	Domestic (*n* = 59)	Imported (*n* = 60)	Domestic (*n* = 70)	Imported (*n* = 37)	Domestic (*n* = 83)	Domestic (*n* = 42)	Domestic & imported (*n* = 30)
*A. baumannii*	7	11	8	4		3	
*A. nosocomialis*	2	7	3	4		1	
*A. pittii*			1				
*A. seifertii*		5		2			
Total	9	23	12	10	0	4	0

**Table 2:** Minimum inhibitory concentration (MIC) distribution of *Acinetobacter* spp. isolates.

Antimicrobial	Test range	Distribution of MICs (μg/mL)											

		≤0.12	≤0.25	≤0.5	≤1	≤2	4≤	8≤	16≤	32≤	64≤	128≤	256≤
Amoxicillin/clavulanic acid 2:1*	2–32						2	15	37	4			
Cefepime	0.25–16			4	19	20	6	5	3	|1			
Cefoxitin*	1–32						2	1	10	45			
Trimethoprim/sulfamethoxazole	0.25–4	22	26	2			4	|4					
Chloramphenicol*	2–64					1	2	1	5	16	30	3	
Ampicillin*	2–64						1	10	25	12	7	3	
Ciprofloxacin	0.12–16	43	12	2	|1								
Streptomycin*	16–128								28	18	7	4	1
Gentamycin	1–64				49	1	5	2	**|**		1		
Ceftazidime	1–16				5	7	31	13	2	|			
Tetracycline	2–128					49	5	1	**|**1		1	1	
Sulfisoxazole*	16–256								38	12			8
Nalidixic acid*	2–128					11	31	13	2				1
Ceftiofur*	0.5–8							28	30				
Meropenem	0.25-4		41	15	1	1		|					
Colistin	2–16					56	|1		1				

Vertical lines in each row indicate the breakpoint of each antimicrobial.

*Indicates no breakpoint in the CLSI guidelines.

**Table 3:** Antimicrobial resistance profile of the strains.

Strain	Sample	Sampling date	Sample origin	Antimicrobial resistance
*Acinetobacter seifertii* KUFSE-ACS004	Imported Beef	2019-02-18	Australia	GEN
*Acinetobacter seifertii* KUFSE-ACS007	Imported Pork	2019-02-18	Spain	TET
*Acinetobacter nosocomialis* KUFSE-ACN017	Domestic Beef	2019-06-17	Chungcheong, Korea	SXT
*Acinetobacter baumannii* KUFSE-ACB021	Domestic Beef	2019-06-17	Chungcheong, Korea	SXT
*Acinetobacter nosocomialis* KUFSE-ACN022	Imported Beef	2019-06-17	Australia	SXT
*Acinetobacter nosocomialis* KUFSE-ACN025	Domestic Pork	2019-06-17	Chungcheong, Korea	FEP, SXT, TET
*Acinetobacter nosocomialis* KUFSE-ACN026	Domestic Pork	2019-06-17	Chungcheong, Korea	SXT, GEN, TET
*Acinetobacter baumannii* KUFSE-ACB029	Imported Beef	2019-07-08	Australia	SXT
*Acinetobacter nosocomialis* KUFSE-ACN033	Domestic Pork	2019-07-08	Gyeonggi, Korea	SXT
*Acinetobacter baumannii* KUFSE-ACB035	Imported Pork	2019-07-08	Spain	SXT
*Acinetobacter nosocomialis* KUFSE-ACN036	Imported Pork	2019-07-08	Netherlands	COL
*Acinetobacter seifertii* KUFSE-ACS055	Imported Beef	2019-09-17	Australia	GEN, COL

*SXT, trimethoprim/sulfamethoxazole; FEP, Cefepime; TET, tetracycline; COL, colistin; GEN, gentamicin

**Table 4:** Results of Resfinder analysis of KUFSE-ACN036.

Colistin						

Resistance gene	Identity	Query/Template length	Contig	Position in contig	Predicted phenotype	Accession number
mcr-4.3	100	1626/1626	ac36_first_contig_40 length 22852 coverage 1846.3 normalized_cov 3.55	12743..14368	Colistin resistance	MG026621

**Table 5:** Top 4 BLAST search results in full part of contig 40 of KUFSE-ACN036.

Description	Max Score	Total Score	Query Cover	E value	Per. Ident	Accession number
*Acinetobacter baumannii* strain EC plasmid pEC_mcr4.3, complete sequence	26,502	27,081	0.65	0	0.99	CP038265.1
*Acinetobacter baumannii* strain EH plasmid pEH_mcr4.3, complete sequence	26,020	26,599	0.63	0	0.9922	CP038261.1
*Acinetobacter baumannii* plasmid pAB18PR065-MCR-4.3, complete sequence	20,740	31,213	0.79	0	0.9953	MK360916.1
*Acinetobacter baumannii* strain MRSN15313 plasmid pAb-MCR4.3, complete sequence	20,740	31,768	0.8	0	0.9953	CP033872.1

**Table 6:** Top 4 BLAST search results on both ends part of contig 40 of KUFSE-ACN036.

Description	Max Score	Total Score	Query Cover	E value	Per. Ident	Accession number
1..6701 of contig 40						
*Acinetobacter baumannii* strain NCIMB 8209 chromosome, complete genome	4303	7087	0.68	0	0.9416	CP028138.1
*Acinetobacter indicus* strain HY20 chromosome, complete genome	4146	7027	0.75	0	0.9321	CP044018.1
*Acinetobacter indicus* strain FS42-2 chromosome, complete genome	4095	6954	0.76	0	0.9283	CP046595.1
*Acinetobacter indicus* strain CMG3-2 chromosome, complete genome	2614	6985	0.74	0	0.9182	CP044445.1
21,131..22,852 of contig 40						
*Acinetobacter baumannii* strain NCIMB 8209 chromosome, complete genome	4303	7087	0.68	0	0.9416	CP028138.1
*Acinetobacter indicus* strain HY20 chromosome, complete genome	4146	7027	0.75	0	0.9321	CP044018.1
*Acinetobacter indicus* strain FS42-2 chromosome, complete genome	4095	6954	0.76	0	0.9283	CP046595.1
*Acinetobacter indicus* strain CMG3-2 chromosome, complete genome	2614	6985	0.74	0	0.9182	CP044445.1
